# Clinical characteristics, CT signs, and pathological findings of Pyrrolizidine alkaloids-induced sinusoidal obstructive syndrome: a retrospective study

**DOI:** 10.1186/s12876-020-1180-0

**Published:** 2020-02-04

**Authors:** Fang Liu, Xinxin Rong, Hui Guo, Dong Xu, Chang Liu, Lingling Meng, Xiaoqian Yang, Tingting Guo, Xuefeng Kan, Yuhu Song

**Affiliations:** 1 Institute of Hematology, Union Hospital, Tongji Medical College, Huazhong University of Science and Technology, Wuhan, 430022 China; 2 Division of Gastroenterology, Union Hospital, Tongji Medical College, Huazhong University of Science and Technology, Wuhan, 430022 China; 3 Institute of Organ Transplantation, Tongji Hospital, Tongji Medical College, Huazhong University of Science and Technology, Wuhan, 430030 China; 4 Department of Infectious Diseases, Tongji Hospital, Tongji Medical College, Huazhong University of Science and Technology, Wuhan, 430030 China; 5 Department of Radiology, Union Hospital, Tongji Medical College, Huazhong University of Science and Technology, Wuhan, 430022 China

**Keywords:** Hepatic sinusoidal obstruction syndrome, Pyrrolizidine alkaloid, Clinical manifestations, Ascites, Histology

## Abstract

**Background:**

One major etiology of hepatic sinusoidal obstruction syndrome (HSOS) in China is the intake of pyrrolizidine alkaloids (PAs). Since PAs-induced HSOS is a rare disease that has not been clearly characterized until now, the aim of this study was to investigate clinical characteristics, CT features, and pathological findings of PA-induced HSOS.

**Methods:**

This retrospective cohort study included 116 patients with PAs-induced HSOS and 68 patients with Budd-Chiari syndrome from Jan 2006 to Sep 2016. We collected medical records of the patients, and reviewed image features of CT, and analyzed pathological findings.

**Results:**

Common clinical manifestations of PAs-induced HSOS were abdominal distention (98.26%), ascites (100%), jaundice (52.94%), abdominal pain (36.36%). Abnormal liver function was observed in most of PAs-induced HSOS. On CT scan, common findings included: ascites, hepatomegaly, the thickening of gallbladder wall, pleural effusion, patchy liver enhancement, and heterogeneous hypoattenuation. Most of the patients had a low ascitic total protein (< 25 g/L) and a high SAAG (≥ 11.0 g/L). In acute stage, pathologic features were massive sinusoidal dilatation, sinusoidal congestion, the extravasation of erythrocytes, hepatocellular necrosis, the accumulation of macrophages, the deposition of hemosiderin. In subacute stage, complete loss of pericentral hepatocytes, sinusoidal dilatation, the deposition of pigment granules were observed.

**Conclusions:**

The PAs-induced HSOS patients displayed distinct clinical characteristics, imaging features, and pathological findings, which provided some evidences for the diagnosis of PAs-induced HSOS.

**Trial registration:**

ChiCTR-DRD-17010709.

## Background

Hepatic sinusoidal obstruction syndrome (HSOS), also known as veno-occlusive disease (VOD), is characterized by damage to sinusoidal endothelium [1, 2]. A central pathogenic event is toxic destruction of hepatic sinusoidal/central venous endothelial cells, and then the sloughed sinusoidal lining cells embolize downstream and obstruct sinusoidal flow [3, 4]. It results in the necrosis of hepatocytes and portal hypertension. In North America and Western Europe, HSOS occurs most commonly in the patients who have received cytoreductive therapy prior to hematopoietic stem cell transplantation (HSCT), or oxaliplatin-containing chemotherapy for colorectal carcinoma [3–6]. While, the intake of pyrrolizidine alkaloids (PAs)-containing herbals or dietary supplement is a major etiology of HSOS in China [7–10]. To date, more than 6000 plant species containing PAs have been identified [11]. One of the most widely used herbals containing PAs is gynura segetum (ie Tsuanqi) in China [12–14].In view of the difference in the etiology of HSOS, clinical profiles and imaging findings of HSOS associated with HSCT or oxaliplatin might be hardly extrapolated to PAs-induced HSOS. In addition, PAs-induced HSOS is a rare disease and clinical manifestations of PAs-induced HSOS resemble other liver diseases. To get a better understanding of PAs-induced HSOS, a large sample of the patients (116 cases) with PAs-induced HSOS and the controls (68 patients with Budd-Chiari syndrome) were enrolled and relevant data were collected. Then, clinical profiles, CT features, and pathological finding of PAs-induced HSOS were investigated in our study.

## Methods

### Study population

**Fig. 1:**
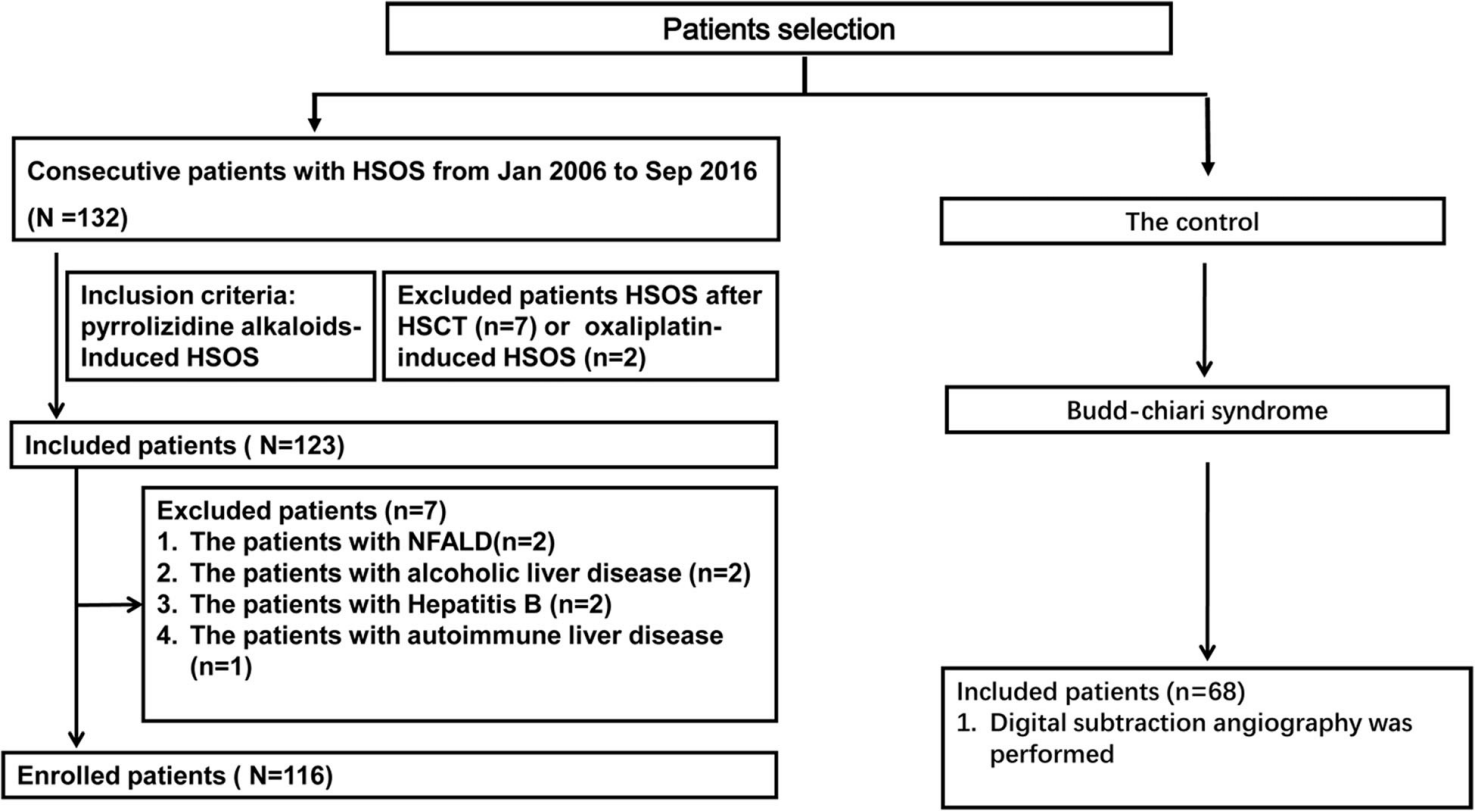
Flowchart of the patients’ enrollment

In this study, we collected 116 patients with PAs-induced HSOS at Union Hospital and Tongji Hospital affiliated to Tongji Medical College, Huazhong University of Science and Technology (HUST) from Jan 2006 to Sep 2016. For PAs-induced HSOS, diagnostic criteria were (i) the patients met the modified Seattle criteria for HSOS (at least 2 of the following: hyperbilirubinemia (> 34.2 μmol/L), hepatomegaly or right upper quadrant pain, ascites and > 2% weight gain due to fluid accumulation) (ii) meeting the criteria for drug-induced liver injury [Roussel Uclaf Causality Assessment Method (RUCAM) score > 5] [15, 16]; (iii) a history of ingestion of PAs [13, 14, 17–20]. A history of ingestion of PAs is essential in defining PAs-induced HSOS. All the enrolled patients ingested PAs-containing *gynura segetum* (ie Tsuanqi) in our study. Other possible etiologies of liver injury, such as viral, alcohol, drug, nonalcoholic fatty liver disease, Budd–Chiari syndrome (BCS), congestive heart diseases, autoimmune liver diseases, were evaluated and excluded carefully (Fig. 1).

PAs-induced HSOS patients should be differentiated from HSCT-related HSOS, or oxaliplatin-induced HSOS. Fortunately, the incidence of HSCT-related HSOS, or oxaliplatin-induced HSOS is very low in China, and medical history provides a definite evidence for differential diagnosis. Importantly, PAs-induced HSOS should be discriminated from Budd-Chiari syndrome (BCS). Thus, 68 patients with BCS were enrolled in our study (Fig. 1). Budd-Chiari syndrome is defined as hepatic venous outflow obstruction at any level from the small hepatic veins (HVs) to the junction of the inferior vena cava (IVC) and the right atrium, regardless of the cause of obstruction [1, 2, 21]. Digital subtraction angiography was performed in the enrolled BCS patients. Approval for this retrospective study was obtained from our college ethics committee, and the requirement for informed consent was waived.

### Data collection

The pertinent data of the patients were extracted from the medical records and tabulated in a database, which included the information about demographic data; presenting symptoms and signs; medical history; laboratory tests (blood test, ascitic examination); pathological changes, therapeutic process.

### Imaging technique and imaging analysis

All CT examinations were performed with one of the following three scanners: 64-detector spiral row scanner (Somatom Definition AS, Siemens, Germany), 32-detector row dual-source CT scanner (Somatom Definition, Siemens, Germany), 320-detector row dynamic volume CT (Aquilion ONE 640, Toshiba, Japan). Contrast-enhanced CT was performed after the injection of contrast medium (iohexol, GE Healthcare Co. Ltd., China; lopromide, Bayer Healthcare Co. Ltd., China; Loversol, Hengrui Medicine Co. Ltd., China). Images were obtained in arterial, portal venous, and equilibrium phases, respectively, with 25–30 s, 55–70 s, and 90–110 s after the injection of contrast medium. All CT images were reviewed in consensus by two experienced observers blinded to clinical data.

### Animal models of PAs-induced HSOS

All animals were maintained under specific pathogen-free conditions at Laboratory Animal Center, Huazhong University of Science and Technology, China. Animals were housed individually in open ventilated cages with distilled water ad libitum until study started. Room temperature was monitored and maintained at 20–26 °C with the light cycle set at 12 h. Monocrotaline (MCT)-treated rats, senecionine-treated mice were used as experimental models of PAs-induced HSOS [22–24]. Male Sprague Dawley rats (weight: 200–220 g) were obtained from Laboratory animal center, Huazhong University of Science and Technology (Wuhan, China). For MCT-treated rats, the rats (*n* = 32) were fasted 12 h, and then the rats were gavaged with monocrotaline (90 mg/kg, Sigma Aldrich, St. Louis, MO, *n* = 16) or PBS (*n* = 16) [22, 25, 26]*.* C57BL/6 male mice were purchased from Beijing Vital River Laboratory Animal Technology Co. Ltd. (Beijing, China). In mouse model of senecionine- induced HSOS, male C57BL/6 mice (8-week-old, *n* = 32) were fasted 12 h, and then the mice were gavaged with senecionine (100 mg/kg, Cayman chem., Ann Arbor, Michigan, *n* = 16) or PBS(*n* = 16) [18, 23, 24, 27]*.* All animals were euthanized by barbiturate overdose. All the animals have received human care in compliance with the institutional animal care guidelines.

### Statistical analysis

Continuous variables were presented as means and standard deviation and categorical variables as numbers and percentage. Independent-samples t-test was used for the analysis of differences between the two groups. The interobserver agreement for assessment of imaging findings was determined using the κ-statistic. The level of agreement was defined as follows: poor, κ < 0.20; fair, −values of 0.2 < κ ≤ 0.40; moderate, 0.4 < κ ≤ 0.60; good, 0.6 < κ ≤ 0.80; and very good, 0.8 < κ ≤ 1.0. A *P* value less than .05 was considered to indicate statistical significance. Statistical analyses were performed using SPSS version 17.0 (SPSS Inc., Chicago, Illinois, USA).

## Results

### Clinical presentation

In this study, 116 patients with a diagnosis of PAs-induced HSOS were enrolled in our study. The mean ages of the PAs-induced HSOS patients were 56.92 ± 12.39 years. In PAs-induced HSOS patients, there were 78 males and 38 females, with a male-to-female ratio of 2.05:1. It indicated that the patients with older age and male gender were frequently affected. Then, clinical manifestations of these patients were evaluated. 98.26% (113/115) of the cases had abdominal distension, a small proportion (36.36%, 40/110) of the PAs-induced HSOS patients had right upper quadrant pain. Meanwhile, our study revealed edema in 39.45% (43/109) of the cases, jaundice in 52.94% (54/102) and weight gain in 15.53% (16/103) of the patients. While, 69.12% (47/68) of the BCS patients had abdominal distension, right upper quadrant pain in 19.12% (13/68) of the cases, edema in 38.24% (26/68) of the cases.

### Laboratory tests (blood routine examination and clinical biochemistry)

Table 1 summarized laboratory tests including blood routine examination and clinical biochemistry at the time of baseline evaluation. Firstly, blood routine examination of the patients was investigated. Generally, the values of erythrocyte and leukocyte were in normal limit in the PAs-induced HSOS patients (Table 1); the value of platelet was lower than the lower limit of normal in 46.81% of the patients. Then, the parameters of clinical chemistry including a panel of liver functional tests and renal function were evaluated. The median levels of liver enzymes (AST, ALT, ALP and GGT) and total bilirubin exceeded upper limit of normal range in the PAs-induced HSOS patients. Simultaneously, synthetic ability of liver was damaged in most of PAs-induced HSOS patients, which was revealed by the abnormality of albumin and PT. While, biomarkers of renal function indicated by urea and creatinine showed that most of the HSOS patients had normal renal function (Table 1). In addition, blood routine examination and clinical biochemistry were analyzed in the controls (BCS patients) and the results showed most of BCS patients had normal blood routine examination and normal liver function, which was demonstrated by liver function test (Table 1).

**Table 1:** Baseline characteristics of the patients and laboratory tests

Variables	PAs-HSOS	BCS	P
Erythrocytes, 10^12^/L	5.00 ± 4.33	4.33 ± 0.84	0.053
Hemoglobin, g/L	135.99 ± 26.81	128.11 ± 24.00	0.061
Leukocyte, 10^9^/L	6.90 ± 2.81	4.87 ± 2.59	<0.001
Platelet, 10^9^/L	114.06 ± 63.48	119.37 ± 83.98	0.651
ALT, U/L	134.5 ± 154.89	40.60 ± 64.75	0.002
AST, U/L	146.31 ± 156.30	49.72 ± 54.81	0.001
ALP, U/L	170 ± 106.89	122.25 ± 69.08	0.003
γ-GT, U/L	160.52 ± 114.56	106.60 ± 85.94	0.002
T-BIL, μmol/L	65.07 ± 78.83	35.58 ± 28.47	0.001
Albumin, g/L	30.71 ± 5.50	35.51 ± 6.29	<0.001
PT, S	17.43 ± 2.64	15.63 ± 2.01	<0.001
Urea, mmol/L	7.19 ± 3.63	5.26 ± 2.76	0.001
Cr, μmol/L	90.65 ± 45.61	64.16 ± 18.67	<0.001

Note: Baseline characteristics of PAs-induced HSOS refer to initial examination during first visit to our hospitals. *PAs-HSOS* PAs-induced HSOS; *BCS* Budd-Chiari syndrome; normal ranges: erythrocytes: 3.0–5.5 × 10^12^/L; hemoglobin: 110–160 g/L; leukocyte: 4–10 × 10^9^ /L; platelet: 100–300 × 10^9^/L; alanine aminotransferase: 5–35 U/L; aspartate aminotransferase (AST):8-40 U/L; alkaline phosphatase (ALP) 40–150 U/L; total bilirubin (T-BIL): 5.1–19 μmol/L; γ-glutamyl transpeptidase (γ-GT) 7–32 U/L; albumin: 35-55 g/L; prothrombin time (PT): 11–16 S; urea: 3.2–7.1 mmol/L; creatinine (Cr): 44–106 μmol/L.

Recently, new EBMT criteria for severity grading of HSOS in adults have been established. In clinical practice, the severity of HSOS is classified by five factors containing bilirubin, liver function enzymes, weight gain, renal function and rate of change [4, 28]. Thus, we evaluated the severity in the PAs-induced HSOS patients using new EBMT criteria. ALT exceeding 8 times the ULN was observed in 13.86% of the cases (Table 2). Besides this, similar pattern was observed in the level of AST, total bilirubin and creatinine (Table 2). Further study showed that 22 (21.15%) patients with mild HSOS, 31(29.81%) patients with moderate HSOS, 21 (20.89%) patients with severe HSOS; 30 (28.85%) patients with very severe HSOS.

**Table 2:** Laboratory tests including blood routine examination and clinical biochemistry at the time of baseline evaluation

Variables	PAs-HSOS	BCS
ALT < 3× ULN, n/N (%)	64.36%(65/101)	94.74%(54/57)
ALT 3–8 × ULN, n/N (%)	21.78%(22/101)	3.51%(2/57)
ALT > 8 × ULN, n/N (%)	13.86%(14/101)	1.75%(1/57)
AST < 3 × ULN, n/N (%)	55.88%(57/102)	91.23%(52/57)
AST 3–8 × ULN, n/N (%)	35.29%(36/102)	8.77%(5/57)
AST > 8 × ULN, n/N (%)	8.82%(9/102)	0
T-BIL <34.2 μmol/L	52.94% (54/102)	61.40% (35/57)
T-BIL 34.2–85.5 μmol/L, n/N(%)	81.37% (83/102)	33.33% (19/57)
T-BIL 85.5–136.8 μmol/L, n/N(%)	7.84%(8/102)	1.75%(1/57)
T-BIL > 136.8 μmol/L, n/N(%)	10.78%(11/102)	3.52%(2/57)
Cr < 1× ULN, n/N(%)	79.52%(66/83)	96.55%(56/58)
Cr 1–2 × ULN, n/N(%)	18.07%(15/83)	3.45%(2/58)
Cr > 2× ULN,n/N(%)	2.41%(2/83)	0

*PAs-HSOS* pyrrolizidine alkaloids-induced hepatic sinusoidal obstruction syndrome, *BCS* Budd-Chiari syndrome, *ALT* alanine aminotransferase, *AST* aspartate aminotransferase, *T-BIL* total bilirubin, Cr creatinine, *ULN* upper limit of normal, *LLN* lower limit of normal

### Ascites analysis

Since ascites is the most common clinical presentation of PAs-induced HSOS, some of the patients received abdominal paracentesis and the results of ascitic fluid analysis were collected. Firstly, we analyzed ascitic fluid total protein (AFTP) and serum-ascites albumin gradient (SAAG). Ascitic fluid total protein (AFTP) belonged to transudate (< 25 g/L) in 74.64% of the patients;100% of the patients had a high SAAG (≥ 11.0 g/L) (Table 3). Secondly, an ascitic fluid cell count and differential were analyzed. An ascitic fluid WBC counts was 134.11 ± 135.62; 100% of the PAs-induced HSOS patients had an ascitic polymorphonuclear (PMN) less than 0.25 × 10^9^/L, which indicated that no ascitic fluid infection occurred in the patients (Table 3). Finally, 41 cases received cytological examination of ascites. The results revealed that lymphocyte and mesothelial cells were main findings in the patients (Table 3). However, only 8 patient with BCS received ascitic fluid analysis in our study and the results of three samples were available, thus the results of ascitic fluid analysis in BCS patients was not shown in our study.

**Table 3:** The results of ascitic fluid analysis

Variable	Number patients with available data	Value
Total protein	71	21.33 ± 7.07
Exudate(≥25 g/L)	71	25.35% (18/71)
SAAG (g/L)	23	18.75 ± 4.79
SAAG(> 11.0 g/L)	23	100% (23/23)
Leukocyte: counts (10^6^/L)	63	134.11 ± 135.62
Polymorphonuclear (10^6^/L)	57	28.50 ± 36.34
Polymorphonuclear≥250 (10^6^/L)	57	0
Cytology	41	
Lymphocyte	41	17.07% (7/41)
Mesothelial cells	41	17.07% (7/41)
Lymphocyte plus mesothelial cells	41	63.41% (26/41)

### Imaging findings of contrast CT in the PAs-induced HSOS patients

**Fig. 2:**
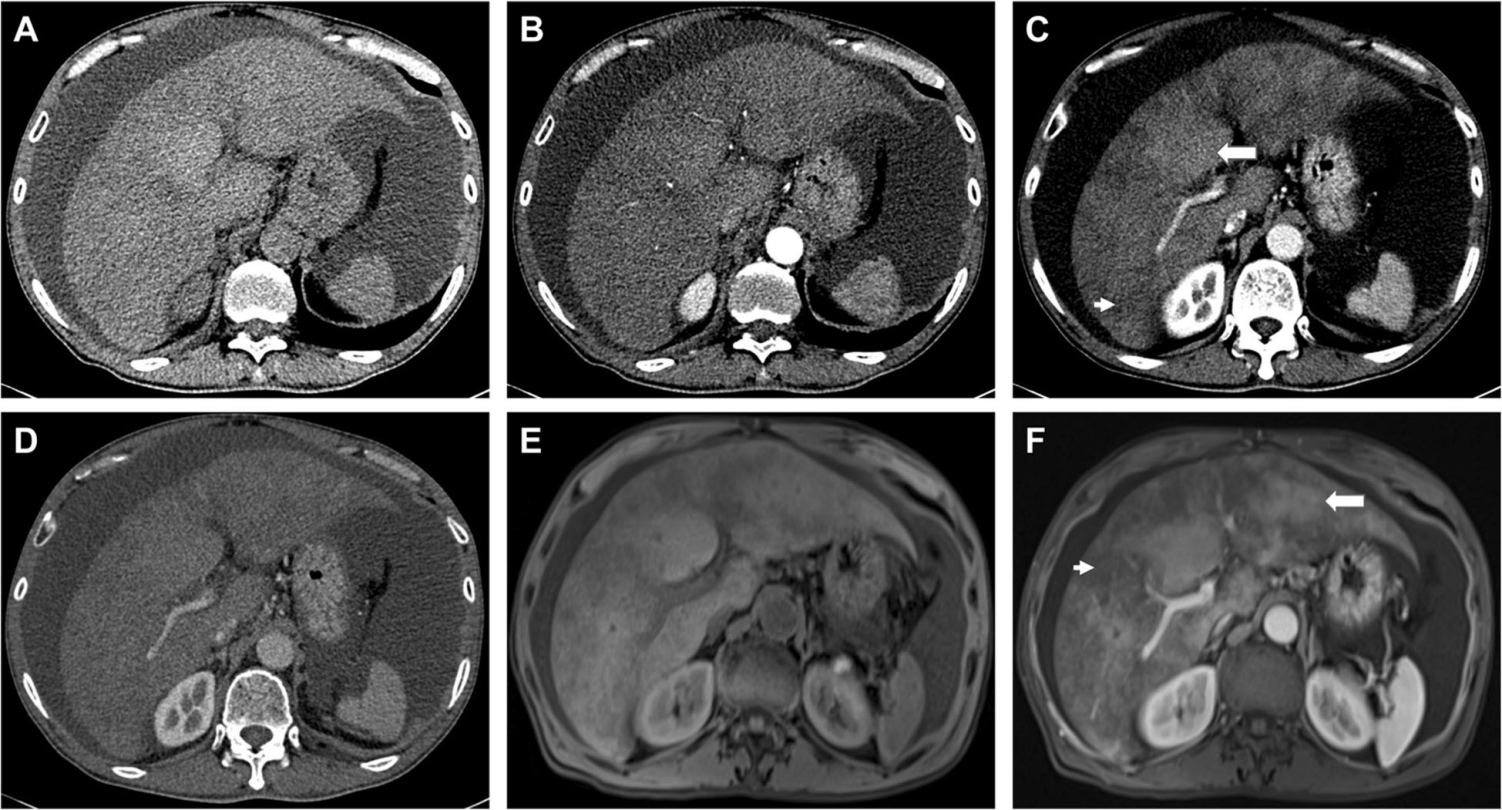
Elderly male patients diagnosed with gynura segetum-induced HSOS received contrast-enhanced CT and MRI scan. **a**-**d** images of plain and contrast-enhanced CT scan; **a** plain CT scan; **b** arterial phase; **c** porta-venous phase; patchy liver enhancement (arrow) and heterogeneous hypoattenuation (arrowhead) were shown; **d** equilibrium phases. **e**-**f**: images of pre-contrast and portal-venous phase on dynamic contrast-enhanced MRI scan;**e** pre-contrast MRI scan; **f** portal-venous phase of MRI scan. Heterogeneous hypointensity (arrowhead) and patchy enhancement (arrow) were shown

Table 4 illustrated radiographic signs seen on contrast-enhanced CT in HSOS patients. Firstly, we determined the findings of pre-contrast CT in the PAs-induced HSOS patients. Ascites, global enlargement of the liver, gallbladder wall thickening, pleural effusion were common signs of PAs-induced HSOS (Table 4); while, a few of the patients (25.32%) had splenomegaly. Secondly, we further analyzed the imaging features of contrast-enhanced CT. On contrast-enhanced CT, patchy liver enhancement (93.67%) and heterogeneous hypoattenuation (100%) in portal-venous phase were two most common radiologic findings (Table 4). Heterogeneous hypoattenuation represented heterogeneous hypoattenuated, or low-density areas; patchy liver enhancement was liver parenchyma adjacent to heterogeneous hypoattenuation appeared inhomogeneous enhancement (Fig. 2). In equilibrium phase of contrast CT, narrowing of right hepatic vein and narrowing of inferior vena cava were common signs (Table 4).

**Table 4:** Summary of radiological features of contrast CT in the patients with PAs-induced HSOS

Variable	Number patients with available data	Value
Hepatomegaly	79	75.95%
Gallbladder wall thickening	77	87.01%
Splenomegaly	79	25.32%
Ascites	79	100%
Pleural effusion	79	68.35%
Regenerative nodules	79	6.33%
Patchy liver enhancement	79	93.67%
Heterogeneous hypoattenuation	79	100%
Hepatic vein narrowing (right branch)	79	94.94%
Narrowing of inferior vena cava	79	87.34%

### Histology

**Fig. 3:**
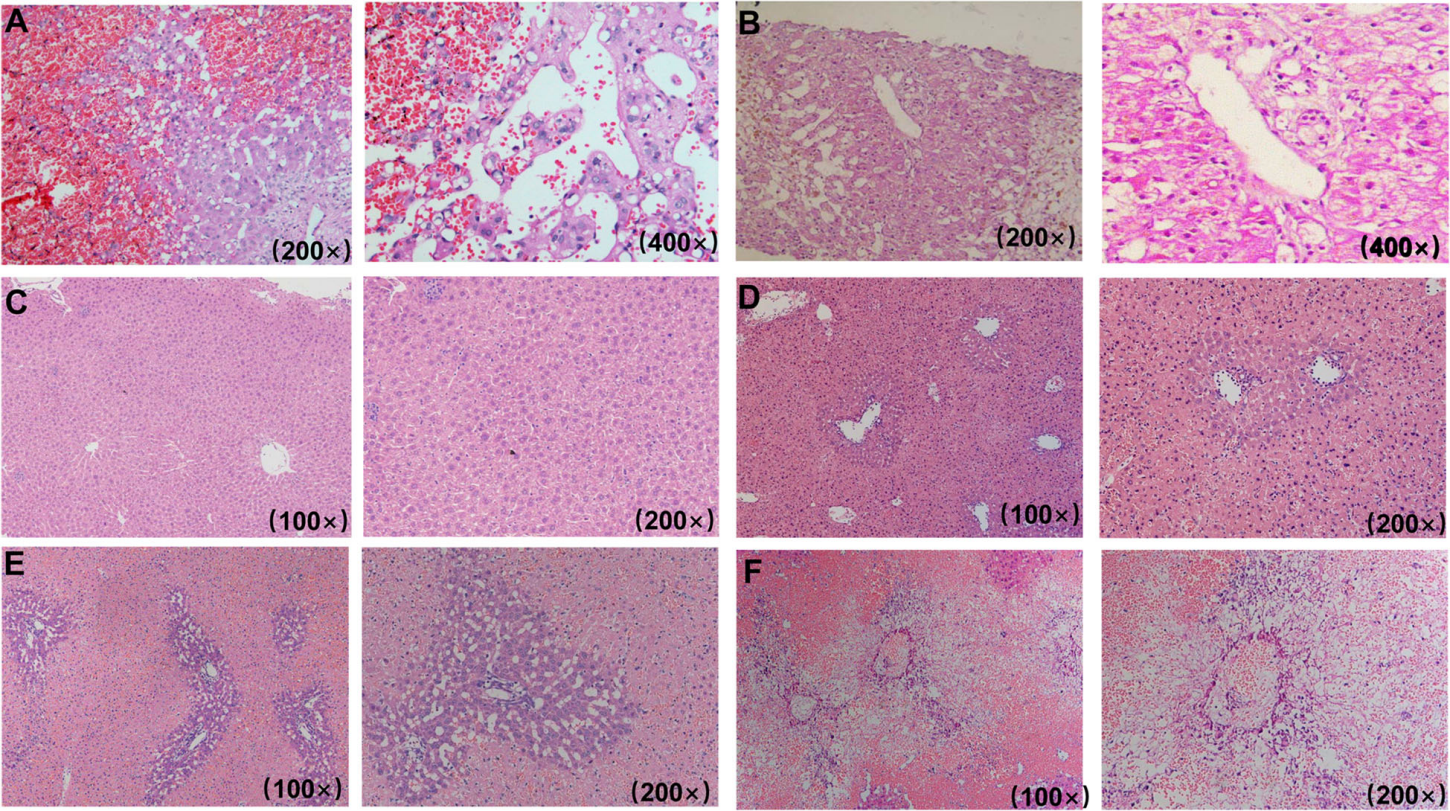
Histology of the patients and rat model of PAs-induced HSOS. **a**: early pathological changes of the PAs-induced HSOS patients; **b**: sub-acute pathological changes of the PAs-induced HSOS patients; **c**: the livers from normal mice were stained by H&E; **d**: 24 h after senecionine administration, the livers from the senecionine-treated mice were stained by H&E; **e**:48 h after monocrotaline administration, the livers from the rats with PAs-induced HSOS were stained by H&E; F: 2 weeks after monocrotaline administration, pathological changes of the rats with PAs-induced HSOS

Most of PAs-induced HSOS patients did not receive percutaneous liver biopsy due to ascites, thrombocytopenia, and coagulation disorders. In addition, the expense and the unavailability of facility confined the application of transjugular biopsy in our hospitals. Thus, only 12 patients received biopsy procedure in our study, and PAs-induced SOS exhibited different pathological features. The patients received liver biopsy within 1–3 months after the initial presentation. The results of liver functional tests at the time of the liver biopsy were shown in Additional file 1: Table S1. Pathological features of the PAs-induced HSOS patients varied from early changes to subacute changes. In acute phase of PAs-induced HSOS (6 cases), liver biopsy showed massive sinusoidal dilatation and sinusoidal congestion predominantly in zone 3 accompanied by the extravasation of erythrocytes into space of Disse. In addition, we found hepatocellular necrosis, the accumulation of macrophages (Fig. 3a). In subacute stage (6 cases), important features of histopathology were the complete loss of pericentral hepatocytes, sinusoidal dilatation, the deposition of hemosiderin derived from destructive erythrocytes (Fig. 3b). The extravasation of erythrocytes and excessive deposition of collagen were not observed (Fig. 3b). The infiltration of inflammatory cells was not obvious in acute and subacute stage.

To confirm the findings, animal models of PAs-induced HSOS were created through the administration of gynura segetum. Seneciphylline, senecionine are major compounds in Gynura segetum [24, 29]; animal models of gynura segetum-induced HSOS should be created through the administration of seneciphylline and/or senecionine. Unfortunately, seneciphylline is not available commercially. Thus, mouse model of gynura segetum-induced HSOS were established through administration of senecionine. 24 h after the administration, pathological features of mouse liver were similar to those of acute HSOS patients (Fig. 3d). Ten days later, senecionine-treated mice recovered by themselves. To mimics the human injury, low-dose senecionine was repeatedly injected into mice in our study. However, most of treated animals died or animal models of PAs-induced HSOS were not established unsuccessfully. Then, PAs-induced HSOS were created through the administration of monocrotaline**.** 48 h after the treatment of monocrotaline, pathological features of rat liver resembled those of acute patients with PAs-induced HSOS (Fig. 3e). At 2 weeks after the administration of monocrotaline, we found the complete loss of pericentral hepatocytes, sinusoidal dilatation and the extravasation of erythrocytes in rat liver, which indicated some of pathological changes in rat model resembled pathological features of subacute PAs-HSOS patients (Fig. 3f). In addition, chronic model of monocrotaline-induced HSOS was not created successfully through repeated administration of low-dose monocrotaline.

### Follow-up

In this study, follow-up data of the patients were collected. At the end of 2-year follow-up, 38% (44/116) of the patients (34 patients lost to follow-up, 10 patients with < 2 years of follow-up) should be excluded; 44 patients survived, 28 patients died. Then, baseline clinical characteristics of the two groups (survival group and death group) were determined and summarized (Table 5). Unfortunately, significant differences were not observed in liver functional tests and renal function between the survival group and the death group. It probably resulted from small sample size. Thus, a prospective multi-center study should be performed to determine the prognosis of the PAs-induced HSOS patients.

**Table 5:** Baseline characteristics of 117 patients with PAs-induced HSOS at the time of diagnosis

Variables	Survival Group	Death Group	*P* Value^*^
Number of patients	44	28	
Age, years	59.00 (47.25–63.00)	61.00 (51.25–65.50)	0.60
Male Sex; *n* (%)	31.00 (70.50%)	20.00 (71.40%)	0.42
Erythrocytes, 10^12^/L	4.58 (4.11–4.86)	4.44 (4.07–4.99)	0.88
Hemoglobin, g/L	140.00 (128.25–149.75)	139.00 (120.00–154.00)	0.84
Leukocyte, 10^9^/L	5.98 (4.59–7.43)	7.51 (5.30–8.73)	0.44
Platelet, 10^9^/L	110.00 (79.00–168.50)	101.00 (73.00–137.00)	0.30
PT, S	16.40 (15.20–18.70)	17.00 (16.20–18.70)	0.53
INR	1.35 (1.23–1.58)	1.41 (1.29–1.60)	0.48
ALT, U/L	68.00 (41.00–147.00)	82.00 (39.00–203.00)	0.35
AST, U/L	88.00 (59.00–166.00)	108.00 (63.00–197.00)	0.40
Total bilirubin, μmol/L	37.25 (21.80–69.98)	39.15 (26.03–122.75)	0.07
ALB, g/l	32.60 (28.20–34.95)	29.85 (27.75–32.18)	0.44
Urea, mmol/L	6.10 (4.12–7.70)	5.83 (4.26–8.77)	0.34
Creatinine, μmol/L	77.50 (64.35–87.32)	81.95 (66.42–97.40)	0.30

Note: Continuous variables are presented as median (25th–75th percentiles), and categorical variables are presented as count (percentage); ^*^ The *p*-values refer to T-test or chi-square test between patients with survival group and death group

## Discussion

Hepatic sinusoidal obstruction syndrome (HSOS) is a rare vascular disease of the liver. Sometimes, HSOS is caused by the intake of PA-containing herbs. To get a better understanding of PAs-induced HSOS, a large sample of the patients (116 cases) with PAs-induced HSOS were enrolled and we analyzed clinical profiles of PAs-related SOS in China. In China, PAs-containing plants were soaked in liquid, such as water, tea, liquor and etc., and the patients took the liquid containing PAs; thus, we did not calculate the dosage of PAs precisely. Give this, we did not determine the correlation between total dosage of PAs and the severity of the diseases. In addition, the duration between the use of Tusanqi and first onset of clinical symptoms ranged from 2 days to 2 years; therefore, we did not distinguish acute stage from subacute/chronic diseases. Some of the patients with first-episode presentation received liver biopsy and the histology showed sub-acute pathological features. The patients with older age and male gender were frequently affected. In China, old people trust the effect of Chinese herbals, so the intake of PAs-containing plants occurred frequently to the patients with older age. Furthermore, metabolic activation of hepatotoxic PAs by cytochrome P450 (CYP) enzymes play a key role in PA-induced liver toxicities. Since hepatic expression of CYP3A1/2 in female rats is lower than that in males, male animals may be more susceptible to PA-induced toxicity than female animals [30–32].

In view of these, we determined clinical manifestations, CT findings, and pathological features of the whole PAs-induced HSOS patients. Common clinical manifestations of PAs-induced HSOS were: ascites (100%), hyperbilirubinemia (52.94%), and hepatomegaly (76%). Meanwhile, a panel of liver function tests demonstrated that most of PAs-induced HSOS patients had abnormal liver function. Importantly, we assessed the severity of PAs-induced HSOS patients for the first time, which is useful to identify the patients requiring early therapeutic intervention. The resulted revealed that most of PAs-induced HSOS patients was in mild or moderate stage, which indicated that most of the patients who received routine treatment had good prognosis. While, clinical manifestations of PAs-induced HSOS mimic other liver disorders, and clinical manifestations did not provide definitive evidence in the diagnosis of PAs-induced HSOS.

In western countries, HSOS occurs most commonly in HSCT, or oxaliplatin -containing chemotherapy, and some studies described their clinical characteristics and pathological features. Previous studies demonstrated weight gain (90%), upper abdominal pain (95%), jaundice (80%) were common clinical manifestations of HSOS following HSCT; only 35% of the patients had ascites [33].In addition, raised ALT, AST and ALP level were observed in the HSOS patients following HSCT [33]. In addition, some researchers reported that clinical manifestations of the patients appear to be mild or absent in the HSOS patients who received oxaliplatin-contained chemotherapy, and the level of ALT, AST, total bilirubin was in normal range [34–36]. Most of the patients were identified through imaging modality and/or histological examination. It indicated that clinical characteristics of HSOS correlated with the etiology of HSOS which was a determinant of the severity in the patients with HSOS.

One of clinical characteristic in PAs-induced HSOS is ascites, thus, we initially collected the results of ascitic fluid analysis. The result showed transudate (< 25 g/L) in 74.64% of the patients and high SAAG (≥ 11.0 g/L) in 100% of the patients. Most of the patients (95–100%) with cardiac ascites had high ascitic fluid total protein concentration (AFTP, ≥25 g/L) [37, 38]. It indicated that ascites in PAs-induced HSOS belonged to portal hypertension-related ascites; diagnostic efficacy of SAAG was superior to that of the exudate-transudate concept. No ascitic fluid infection occurred in PAs-induced HSOS patients, which revealed by ascitic fluid PMN counts.

The common features of dynamic CT in the PAs-induced HSOS patients contained: ascites, hepatomegaly, gallbladder wall thickening, pleural effusion, hepatic vein narrowing, patchy liver enhancement, and heterogeneous hypoattenuation. In HSOS patients after HSCT, hematomegaly, periportal edema, ascites, and narrowing of right hepatic vein were common findings of CT [39, 40]. Heterogeneity of liver parenchyma was observed in the patients of colorectal cancer who had recieved oxaliplatin-based chemotherapy [34, 41]. All these indicated that the disparity in imaging findings of contrast-enhanced CT attributed to the etiology in the patients with HSOS. The etiology of HSOS is an important determinant of the severity, which may result in the difference in radiologic findings of contrast-enhanced CT. More importantly, CT sign of Budd-Chiari syndrome was analyzed, imaging features of BCS patients were described in our previous study [14]. To avoid self plagiarism, imaging signs were not provided in our manuscript.

Histological examination provided a definitive evidence of PAs-induced HSOS. However, liver biopsy was invasive and difficult to perform in routine practice due to thrombocytopenia, clotting abnormalities and extensive ascites. We firstly demonstrated PAs-induced HSOS exhibited different pathological features upon different phases. In acute stage, sinusoidal congestion, sinusoidal dilation, the necrosis of hepatocytes and the extravasation of erythrocytes in zone 3 were the characteristics of PAs-induced HSOS. In addition, macrophages infiltrated into the space of Disse, and engulfed erythrocytes. Sequentially, macrophages degraded the hemoglobin in erythrocytes, producing hemosiderin. Thus, important pathological changes in PAs-induced HSOS was the infiltration of macrophages and the deposition of hemosiderin. In sub-acute stage, pathologic examination showed complete loss of pericentral hepatocytes, sinusoidal dilatation, the deposition of pigment granules. Thus, it indicated that histological findings of PAs-induced HSOS were variable, depending upon the stages and the severity. In addition, different phases of pathological changes (early changes vs subacute changes) were observed when the patients shared similar periods from onset of illness. The varieties of pathological manifestations depend on age, the PAs dose, the period, and individual variation.

In clinical practice, PAs-induced HSOS should be considered when the patients presented with abdominal distension, jaundice, ascites, hepatomegaly. Firstly, a detailed drug-use history should be obtained. The detection of pyrrole protein adducts (PPAs) is essential for a diagnosis of PA-induced HSOS when PAs exposure is obscure. Then, dynamic enhanced-contrast CT scanning and/or MRI examination should be performed in all suspected patients. If typical signs of PA-induced HSOS are discovered, then the diagnosis can be confirmed [13, 14, 19, 42, 43]. Other chronic liver diseases, such as BCS, decompensated cirrhosis, cardiac insufficiency, should be excluded. In guideline of pyrrolizidine alkaloid-induced HSOS issued by Chinese Society of Gastroenterology Committee, supportive symptomatic treatment is the basic PA–HSOS treatment regimen. Anticoagulant therapy should be started as soon as possible in acute/subacute stage patients after ruling out contraindications TIPS should be considered when the patients do not respond to medical treatment [42]. Public education programs on potential harms of PAs-containing plants should be developed through five media (newspapers, TV, the internet, radio, and magazines). Thus, the ingestion of PA-containing plants should be prohibited due to the toxicity of PAs.

Obviously, our study had several limitations. Firstly, it was retrospective study, not prospective study. Selection bias occurred in retrospective cohort studies. Secondly, liver biopsy was not performed in most of the patients; therefore, histological evidences were not available in most of the patients. A small sample of liver biopsy, sampling variability and patchy distribution might result in the bias. Thirdly, some of the relevant data of the patients were not available. Finally, follow-up data could not be provided since retrospective study involved 11-year span.

## Conclusions

In conclusion: the PAs-induced HSOS patients displayed distinct clinical characteristics, imaging signs, and pathological features compared with HSOS associated with HSCT and oxaliplatin-containing chemotherapy. Further studies should be performed to explore histological changes based on large samples, develop suitable therapeutic strategies, and investigate the prognosis in future.

## Supplementary information

**Additional file 1: Table S1.** The results of liver functional test at the time of the liver biopsy

## Data Availability

The raw data generated and analyzed in the current study are not publicly available due to appropriate protection of patient personal information but are available from the corresponding author on reasonable request.

## Abbreviations

AFTP: Ascitic fluid total protein
ALP: Alkaline phosphatase
ALT: Alanine aminotransferase
AST: Aspartate aminotransferase
BCS: Budd-Chiari syndrome
CT: Computed tomography
DCE: Dynamic contrast-enhanced
HSCT: Hematopoietic stem cell transplantation
HSOS: Hepatic sinusoidal obstruction syndrome
LLN: Lower limit of normal
MRI: Magnetic resonance imaging
PA: Pyrrolizidine alkaloid
PMN: Polymorphonuclear
PT: Prothrombin time
SAAG: Serum ascites albumin gradient
ULN: Upper limit of normal
VOD: Veno-occlusive disease
WBC: White blood cell
γ-GT: γ-glutamyl transpeptidase
