# Supplementary material for: Clinical characteristics, CT signs, and pathological findings of Pyrrolizidine alkaloids-induced sinusoidal obstructive syndrome: a retrospective study
Source: BMC Gastroenterol. 2020 Feb 4;20:30. doi: 10.1186/s12876-020-1180-0 (PMC7001201; doi:10.1186/s12876-020-1180-0)
Supplement: Supplementary file 1 — Additional file 1: Table S1. The results of liver functional test at the time of the liver biopsy [file 12876_2020_1180_MOESM1_ESM.docx]

**Supplementary data**

**Clinical characteristics, CT Signs, and Pathological Findings of Pyrrolizidine Alkaloids-induced Sinusoidal Obstructive Syndrome: a retrospective study**

Fang Liu^1^, Xinxin Rong ^2^, Hui Guo^3^, Dong Xu ^4^, Chang Liu^2^, Lingling Meng ^2^, Xianqian Yang^2^, Tingting Guo^5^, Xuefeng Kan^5^, Yuhu Song^2^

^1^Institute of Hematology, Union Hospital, Tongji Medical College, Huazhong University of Science and Technology, Wuhan 430022, China;

^2^ Division of Gastroenterology, Union Hospital, Tongji Medical College, Huazhong University of Science and Technology, Wuhan 430022, China;

^3^Institute of Organ Transplantation, Tongji Hospital, Tongji Medical College, Huazhong University of Science and Technology; Wuhan 430030, China;

^4^Department of Infectious Diseases, Tongji Hospital, Tongji Medical College, Huazhong University of Science and Technology; Wuhan 430030, China;

^5^Department of Radiology, Union Hospital, Tongji Medical College, Huazhong University of Science and Technology, Wuhan 430022, China.

**Table.S1**.the results of liver functional test at the time of the liver biopsy.

| Variables | Number patients with available data | Value |
| --- | --- | --- |
| ALT, U/L | 11 | 94.36±123.57 |
| AST, U/L | 12 | 83.42±49.67 |
| ALP, U/L | 11 | 208.82±105.73 |
| γ-GT, U/L | 11 | 167.55±113.68 |
| T-BIL, μmol/L | 11 | 101.23±161.38 |
| Albumin, g/L | 11 | 31.46±7.04 |

Normal ranges: alanine aminotransferase (ALT) : 5-35 U/L; aspartate aminotransferase (AST):8-40U/L; alkaline phosphatase (ALP) 40-150 U/L; γ-glutamyl transpeptidase (γ-GT) 7-32 U/L; total bilirubin (T-BIL): 5.1-19 μmol/L; [albumin](file:///D:\Program%2520Files\Youdao\Dict\6.3.69.5012\resultui\frame\javascript:void(0);): 35-55g/L.
